# Effect of Aqueous Extract of Phenolic Compounds Obtained from Red Wine in Experimental Model of Colitis in Mice

**DOI:** 10.3390/cimb44060188

**Published:** 2022-06-18

**Authors:** Vanessa Mateus, João Estarreja, Inês Silva, Fernando Gonçalves, Edite Teixeira-Lemos, Rui Pinto

**Affiliations:** 1 H&TRC—Health and Technology Research Center, ESTeSL—Lisbon School of Health Technology, Polytechnic Institute of Lisbon, 1990-096 Lisbon, Portugal; vanessa.mateus@estesl.ipl.pt (V.M.); joaoestarreja@edu.ulisboa.pt (J.E.); ines.silva@estesl.ipl.pt (I.S.); 2 iMed.ULisboa, Faculty of Pharmacy of the University of Lisbon, 1600-277 Lisbon, Portugal; 3 Higher Agricultural School of Viseu—Polytechnic Institute of Viseu, 3500-606 Viseu, Portugal; fgoncalves@esav.ipv.pt (F.G.); etlemos2@gmail.com (E.T.-L.); 4 CERNAS Research Centre, Polytechnic Institute of Viseu, 3504-510 Viseu, Portugal; 5 JCS, Joaquim Chaves, Clinical Laboratory, 1495-068 Lisbon, Portugal

**Keywords:** polyphenols, red wine, colitis, inflammation, inflammatory bowel disease, TNBS-induced colitis

## Abstract

Background: Inflammatory bowel disease (IBD) is a chronic relapsing inflammatory disorder represented by Crohn’s disease and ulcerative colitis. Currently, there is no cure and pharmacological treatment aims to induce and maintain remission on patients. Because the therapy reveals a relatively high toxicity, during a long-term utilization, it is essential to investigate new pharmacological approaches. Polyphenols, commonly present on red wine, have shown health-beneficial effects related to their antioxidant and anti-inflammatory effects through the inhibition of NF-kB activation, COX-2 and iNOS induction. In this sense, it would be interesting to study their effects in an IBD context. Therefore, this study aims to evaluate the effects of an aqueous extract of phenolic compounds in a 2,4,6-Trinitrobenzenesulfonic acid (TNBS)-induced model of colitis. Method: Experimental colitis was induced in mice through an intrarectal administration of TNBS and then the mice were treated with an aqueous extract of phenolic compounds intraperitoneally for four days. Results and Discussion: The extract demonstrated an anti-inflammatory effect, reducing TNF-α levels in the colon, and had a beneficial effect on the extraintestinal manifestations related to IBD, without any significant side effects. The extract of phenolic compounds demonstrated to be a valuable object of study for the management of IBD in the future.

## 1. Introduction

Inflammatory bowel disease (IBD) is a chronic inflammatory pathology characterized by an immune system activation, which can be categorized in two phenotypes: Chron’s disease and ulcerative colitis [1,2,3,4,5]. Both conditions are autoimmune disorders that are not medically curable [5]. IBD is predominantly observed in developed countries, but with an increasing incidence and prevalence in other regions worldwide [6,7]. Indeed, it is estimated that approximately seven million people currently live with IBD [8]. In terms of gastrointestinal signs and symptoms, it can be emphasized the presence of diarrhea, abdominal pain, and hematochezia. Additionally, it also can develop extraintestinal manifestations such as anemia, fever, weight loss, arthritis, sclerosing cholangitis, uveitis, pyoderma gangrenosum and erythema nodosum [1,9]. Currently, the therapeutic strategies for patients with IBD are related to modifications in lifestyle habits, pharmacological treatments and surgical treatments. Indeed, the pharmacological approaches include aminosalicylates, corticosteroids and immunomodulators, which represent the first-line therapy. Other drugs, such as antibiotics, probiotics and biological agents, provide an alternative therapy. However, there is no curative treatment strategy and, due to several side effects demonstrated by a long-term use of these drugs, it is essential to investigate new potential pharmacological approaches [1,10,11,12].

Polyphenols have demonstrated several health-beneficial effects related to their antioxidant activity in the context of preventing oxidative stress-induced pathologies [13]. Among the biological properties observed from polyphenols, the antioxidant, anti-inflammatory and antimicrobial effects can be emphasized [1,13,14,15,16]. Anthocyanins, a major class of polyphenols, are one of the most important health-promoting dietary supplements and are widely distributed in the human diet [1]. Indeed, epidemiological studies have reported that anthocyanins may contribute to chemopreventive activities of several chronic inflammatory diseases [17]. The polyphenols present in red wine may change the gut microbial composition by their antimicrobial properties, affecting their functional relations with the host [18]. Among the major wine phenolic compounds that may reach the gut, polymeric flavan-3-ols or pro-anthocyanins have shown the capability of promoting the growth of beneficial bacteria and the inhibition of pathogenic bacteria [19]. Thus, the inhibitory effects of red wine on inflammatory mechanisms, such as NF-kB activation, IL-8 production, COX-2 and inducible nitric oxide synthase induction, provide evidence that polyphenols are able to ameliorate or prevent intestinal inflammation [13]. Currently, there is already preclinical data that suggest a beneficial role of several specific bioactive compounds present on wine, mainly resveratrol, in several inflammatory-related disorders [20,21]. However, there is a lack of evidence concerning what the exact role of a more complete extract, including different phenolic compounds present on red wine, in an IBD context might be. 

According to the information above, we hypothesize that the administration of an extract of phenolic compounds obtained from red wine, in the context of IBD, will demonstrate a significant anti-inflammatory effect. In this sense, this study aims to evaluate the efficacy and safety of an aqueous extract of phenolic compounds prepared from red wine in a 2,4,6-Trinitrobenzenesulfonic acid (TNBS)-induced acute mouse model of colitis. The results from this experiment can be a valuable source of information for the scientific community concerning the role of a complex interaction between several bioactive compounds, namely polyphenols, in the management of inflammatory-related disorders. 

## 2. Materials and Methods

### 2.1. Chemicals

2,4,6-Trinitrobenzene sulfonic acid (TNBS 5%) was bought from Sigma Chemical (Sintra, Portugal). Ketamine (Imalgene^®^ 1000) and Xilazine (Rompun^®^ 2%) were acquired from Merial (Lisbon, Portugal) and Bayer (Lisbon, Portugal), respectively. The phenolic compounds extract (PCE) was provided from Instituto Politécnico de Viseu, by Escola Superior Agrária de Viseu, Viseu, Portugal. ADVIA^®^ kit came from Siemens Healthcare Diagnostics (Erlangen, Germany). ELISA assay kit for tumor necrosis factor-α (TNF-α) measurements was acquired from Hycult Biotechnology (Uden, The Netherlands).

### 2.2. Animals

Forty-six CD-1 mice (male, 10 weeks old, 21–31 g body weight) were used for the experiment. The animals were housed in standard polypropylene cages, with access to food and water ad libitum. In terms of macroenvironment, mice were kept under a uniform and controlled temperature between 18–23 °C, a humidity oscillating between 40–60% and had a 12 h light/dark cycle. Animal care was in strict accordance with the Declaration of Helsinki, EEC Directive of 24 November 1986 (nº 86/609/EEC), the relevant Portuguese laws (D.R. nº 31/92 and D.R. 153 I-A 67/92) and all subsequent legislation. The experiment was approved by the Ethics Committee for Animal Experimentation of the Faculty of Pharmacy, University of Lisbon (code 35/2014). The experiment took place in a pharmacology laboratory in the Faculty of Pharmacy at the University of Lisbon.

### 2.3. Induction of Experimental Colitis

The mice were left unfed during the 24 h prior to induction. First, on induction day, mice were anesthetized with 40 μL of a mixture of ketamine 100 mg/kg + xylazine 10 mg/kg by an intraperitoneal (IP) injection. TNBS was instilled through a single intracolonic administration [22,23,24]. The technique consisted of the insertion of a cannula into the colon, such that the tip was 4 cm proximal to the anus, and then 100 μL TNBS 2.5% was administrated. Finally, mice were kept in a Trendelenburg position for 1 min to avoid reflux.

### 2.4. Experimental Design

In order to evaluate the efficacy and safety of the administration of PCE in the TNBS-induced model of colitis, the animals were divided into different groups, such as: TNBS group (*n* = 6)—control group, wherein mice were only induced with colitis through the administration of TNBS, as previously described; TNBS + PCE100 (*n* = 10) and the TNBS + PCE500 (*n* = 10) groups—experimental groups, wherein animals were induced with colitis through the administration of TNBS and treated daily with 100 mg/kg and 500 mg/kg of PCE by IP administration for four days, respectively; PCE500 group (*n* = 10)—control group, wherein mice were treated daily with only 500 mg/kg of PCE by IP administration for four days; ethanol group (*n* = 5)—control group, wherein mice received a single intracolonic administration of 100 μL of the TNBS’s vehicle, which was 50% ethanol; sham group (*n* = 5)—control group, wherein mice received a single intracolonic administration of 100 μL of 0.9% NaCl. 

On the fourth day, which corresponded with the end of the experiment, the animals received another IP administration of 40 μL of the ketamine 100 mg/kg + xylazine 10 mg/kg mixture in order to be anesthetized. Then, a cardiac puncture was carried out in each mouse to collect the blood samples. Afterwards, the animals suffered a cervical dislocation and the colon tissue was divided, detached and freed from adjacent tissues. At the end, the colon tissue was washed with phosphate-buffered saline.

### 2.5. Preparation and Characterization of Phenolic Compound Extract

In order to develop the PCE for the IP administration, it was necessary to prepare an aqueous extract, which was obtained by hot extraction. The procedure used to obtain the extract was as follows: weigh 1.5 g of phenolic compounds from red wine; place the powder obtained in cooled, previously boiled water and magnetically stir for 30 min; centrifugate (3500 rpm for 15 min) the resulting aqueous extract; freeze the supernatant. Posteriorly, through high-performance liquid chromatography, it was possible to clearly quantify the total and detailed phenolic content, expressed as milligram of gallic acid equivalents (GAE) per liter of extract. 

### 2.6. Monitoring of Clinical Signs

Mice were observed and monitored daily in terms of body weight, morbidity, consistency of feces, hematochezia and anus appearance.

### 2.7. Biological Markers

Upon collecting the blood samples, they were centrifuged (2000 rpm for 15 minutes at 6 °C) to analyze several biochemical markers, such as alkaline phosphatase (ALP), alanine aminotransferase (ALT), urea and creatinine. Throughout the process of analysis of the biochemical markers, an automated clinical chemistry analyzer (ADVIA^®^1200) was utilized. Additionally, the fecal hemoglobin was evaluated through a quantitative method by immunoturbidimetry (Kroma Systems^®^). Finally, the colon length was also analyzed using a measuring scale.

### 2.8. Analysis of the Inflammatory Response

To evaluate the inflammatory response, a pro-inflammatory cytokine, TNF-α, was measured and expressed as pg/ml. Samples of colonic tissue were weighed and homogenized (Ultra-turrax T25, 13.500 rev/min, twice for 30 s) in phosphate-buffered saline. Afterwards, the samples were centrifuged (15.000 rpm for 15 min at 4 °C). The aliquots of the supernatant were maintained at −20 °C until evaluation. Then, the cytokine level was measured at 450 nm, through a spectrophotometric method (ELISA kit Quantikine, Hy-cult Biotechnology, Uden, The Netherlands).

### 2.9. Data Processing and Statistical Analysis

All results were expressed as the mean ± SD of N observations, where N represents the number of animals studied. Data analysis was performed by using GraphPad Prism 7.0 software (GraphPad, San Diego, CA, USA). In order to conclude if the data follow a normal distribution, the D’Agostino–Pearson normality test was performed. The results were then analyzed by one-way ANOVA to determine statistical significance between the experimental and control groups, followed by Tukey’s post hoc test for multiple comparisons. A *p*-value of less than 0.05 was considered significant.

## 3. Results

### 3.1. Composition of Phenolic Compounds Extract

The PCE was composed of 1323 ± 37 (mg/L GAE) of total phenolic compounds, 1.17 ± 0.18 (g/L) of tannins and 234.4 ± 4.1 (mg/L Mv3Glc) of total anthocyanins. Additionally, it contained a total amount of 465.5 ± 30 mg/L of sugars and 98.6 ± 6.6 mg/L of amino acids. Detailed analysis also showed the presence of 11.1 mg/L of monomeric procyanidins, 72.7 mg/L of oligomeric procyanidins, 28.6 mg GAE/L of benzoic acids, 46.9 mg GAE/L of hydroxycinnamic acids, 15.2 mg/L of catechin and 375.3 mg/L of monomeric anthocyanins.

### 3.2. Manifestation of Clinical Signs

The animals present in the TNBS group presented changes of intestinal motility, characterized by diarrhea or soft stools and moderate edema of the anus. In relation to the PCE-treated groups, taking into account both dosages, some mice also presented alterations in intestinal motility, but in a lighter form. The control groups, as PCE500, ethanol and sham groups, remained without any alterations.

**Figure 1:**
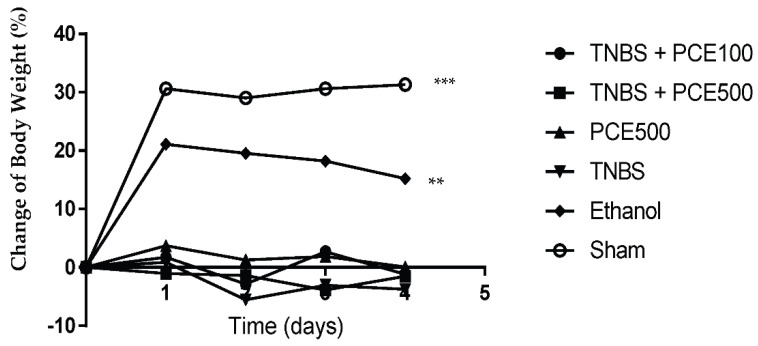
Change of body weight during PCE treatment in the IBD. The results are represented as mean ± SD [*n* (TNBS + PCE100) = 10; *n* (TNBS + PCE500) = 10; *n* (PCE) = 10; *n* (TNBS) = 6; *n* (Ethanol) = 5; *n* (Sham) = 5; ** *p* < 0.01; *** *p* < 0.001 compared with TNBS group, Two-way ANOVA followed by Tukey’s post hoc test]. Abbreviations: IBD, inflammatory bowel disease; PCE, phenolic compounds extract; TNBS, 2,4,6-Trinitrobenzene sulfonic acid.

Regarding body weight changes (Figure 1), the TNBS group presented a slight decrease in body weight over the experimental period (−2.3 ± 2.9%). The results of body weight changes between TNBS group and PCE treatment groups, at both dosages, were similar. At the end of the experimental period, the TNBS + PCE100 group presented a variation of 0.08 ± 0.3% of its initial weight and the TNBS + PCE500 group presented a variation of −1.58 ± 1.9%. The PCE500, ethanol and sham groups gained 1.4 ± 2.2%, 14.8 ± 8.5% and 24.3 ± 13.6% of their initial weights, respectively, but this increase was only significant in the ethanol and sham groups compared to the TNBS group (*p* < 0.01 and *p* < 0.001, respectively).

### 3.3. Biological Markers

#### 3.3.1. Colon Length

**Figure 2:**
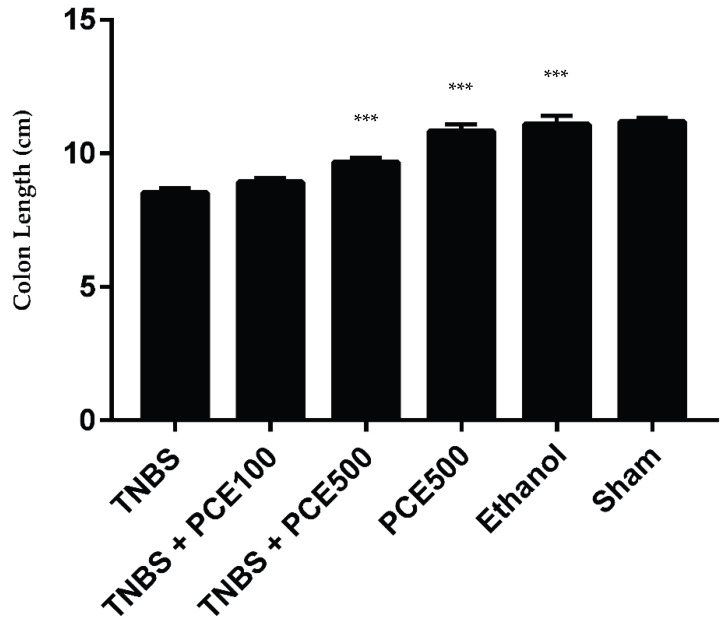
Effect of PCE treatment on colon length in the IBD. The results are represented as mean ± SD [*n* (TNBS + PCE100) = 10; *n* (TNBS + PCE500) = 10; *n* (PCE) = 10; *n* (TNBS) = 6; *n* (ethanol) = 5; *n* (sham) = 5; *** *p* < 0.001 compared with TNBS group, one-way ANOVA followed by Tukey’s post hoc test]. Abbreviations: IBD, inflammatory bowel disease; PCE, phenolic compounds extract; TNBS, 2,4,6-Trinitrobenzene sulfonic acid.

As a marker of tissue integrity, the colon’s length was evaluated and compared between all experimental groups (Figure 2). The mice from TNBS group had a significant shortening of colon length (8.5 ± 0.5 cm) relative to the sham group (11.2 ± 0.5 cm), as a comparable normal colonic tissue (*p* < 0.001). The PCE treatment had no significant effect on the colon length, whereas the TNBS+PCE100 (8.9 ± 0.6 cm) and TNBS+PCE500 (9.7 ± 0.6 cm) groups revealed similar results to TNBS group. The PCE500, ethanol and sham groups presented similar results between them, measuring 10.8 ± 1 cm, 11.1 ± 0.7 cm and 11.2 ± 0.2 cm, respectively.

#### 3.3.2. Fecal Hemoglobin

**Figure 3:**
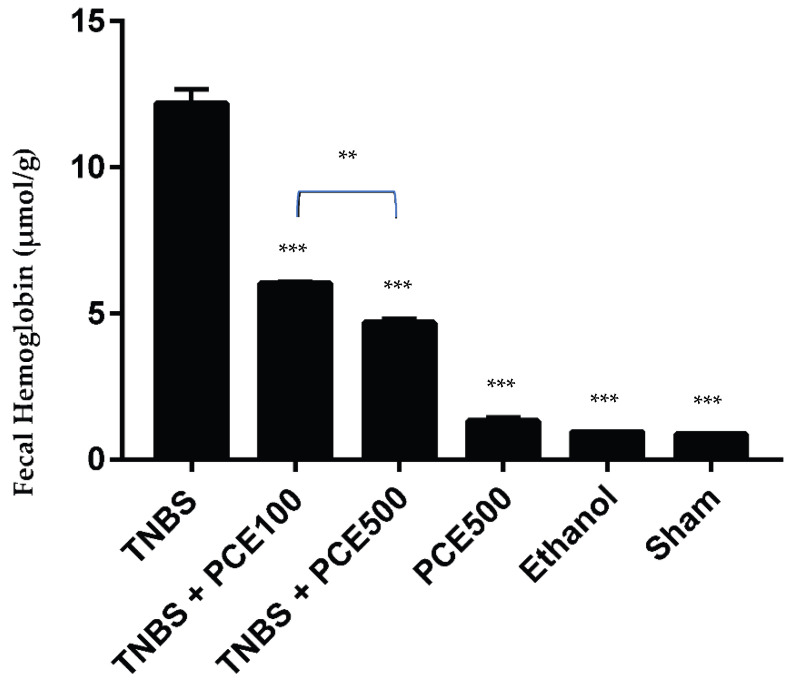
Effect of PCE treatment on fecal hemoglobin in the IBD. The results are represented as mean ± SD [ *n* (TNBS + PCE100) = 10; *n* (TNBS + PCE500) = 10; *n* (PCE) = 10; *n* (TNBS) = 6; *n* (Ethanol) = 5; *n* (Sham) = 5; ** *p* < 0.01; *** *p* < 0.001 compared with TNBS group, one-way ANOVA followed by Tukey’s post hoc test]. Abbreviations: IBD, inflammatory bowel disease; PCE, phenolic compounds extract; TNBS, 2,4,6-Trinitrobenzene sulfonic acid.

The fecal hemoglobin was measured and compared between all groups, which allowed the evaluation of the intensity of the hemorrhagic focus and the impact of PCE treatment (Figure 3). The concentration of fecal hemoglobin in the TNBS group was increased compared to the sham group (12.2 ± 1.2 vs. 0.9 ± 0.1 μmol/g feces, *p* < 0.001). PCE treatment had a statistically significant influence on the intensity of hemorrhagic focus a significant difference in fecal hemoglobin was also observed between the TNBS group and both PCE dosages, namely the TNBS+PCE100 group (6.0 ± 0.3 μmol/g feces, *p* < 0.001) and the TNBS+PCE500 group (4.7 ± 0.6 μmol/g feces, *p* < 0.001). Regarding the differences between dosages of PCE, a dose-dependent effect (*p* < 0.01) was observed because fecal hemoglobin decreased when PCE dose was increased. Mice from PCE500 (1.3 ± 0.6 μmol/g feces) and ethanol groups (0.95 ± 0.1 μmol/g feces) did not display significant changes compared to the sham group (0.9 ± 0.1 μmol/g feces).

#### 3.3.3. Alkaline Phosphatase

**Figure 4:**
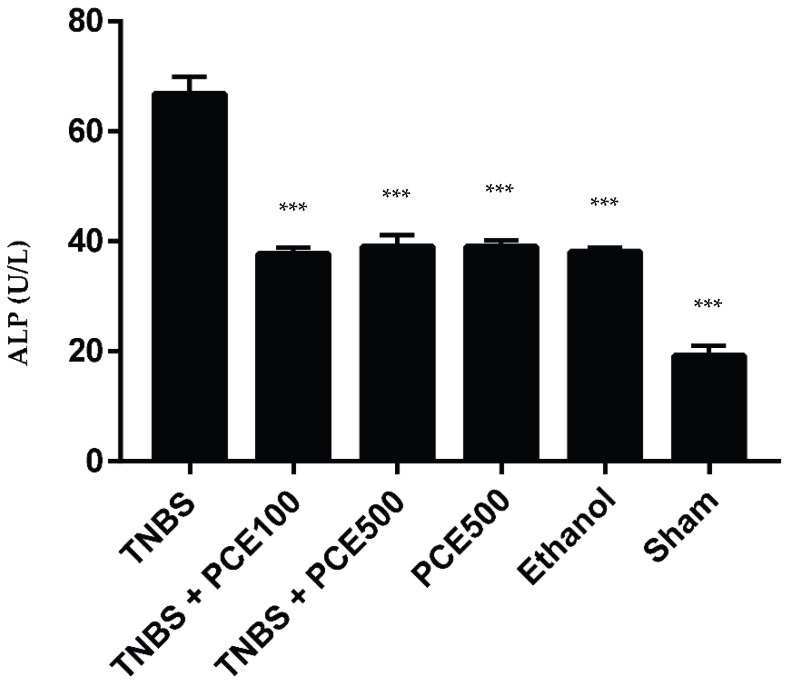
Effect of PCE treatment on total ALP concentration in the IBD. The results are represented as mean ± SD [ *n* (TNBS + PCE100) = 10; *n* (TNBS + PCE500) = 10; *n* (PCE) = 10; *n* (TNBS) = 6; *n* (Ethanol) = 5; *n* (Sham) = 5; *** *p* < 0.001 compared with TNBS group, one-way ANOVA followed by Tukey’s post hoc test]. Abbreviations: ALP, alkaline phosphatase; IBD, inflammatory bowel disease; PCE, phenolic compounds extract; TNBS, 2,4,6-Trinitrobenzene sulfonic acid.

Intestinal ALP concentrations were measured and compared between all groups (Figure 4). The ALP concentration on the TNBS group was statistically higher when compared with the sham group (66.8 ± 7.6 vs. 19.2 ± 4.0 U/L, *p* < 0.001). The PCE treatment was able to decrease the ALP concentration at both dosages, an effect which had statistical significance (*p* < 0.001 compared with TNBS group). However, the differences between dosages of PCE were not statistically significant; the TNBS + PCE100 group revealed 37.7 ± 3.8 U/L, whereas the TNBS+PCE500 group presented 39.0 ± 6.6 U/L. The ALP in the PCE500 and ethanol groups was increased compared with sham group (39.0 ± 3.5 U/L and 38.0 ± 1.8 U/L, respectively).

#### 3.3.4. Renal Function

**Figure 5:**
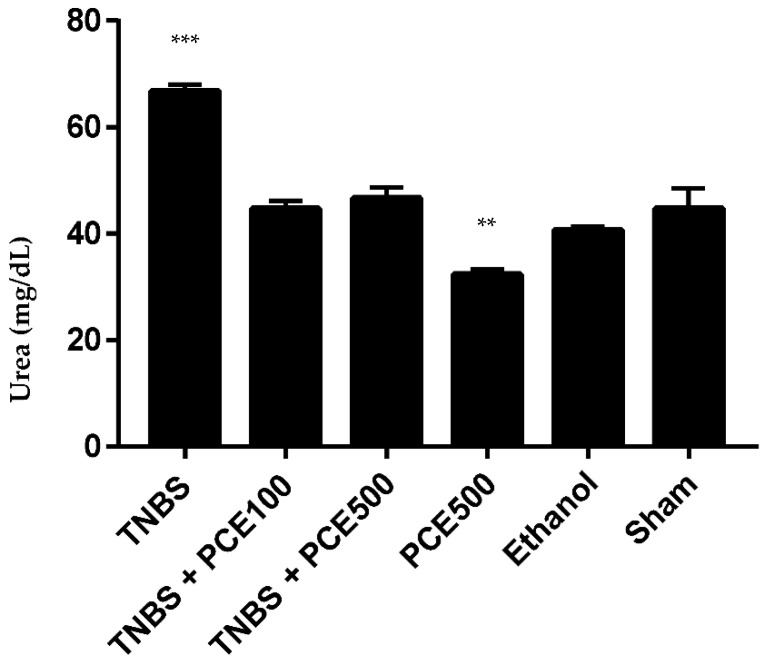
Effect of PCE treatment on urea concentration in the IBD. The results are represented as mean ± SD [ *n* (TNBS + PCE100) = 10; *n* (TNBS + PCE500) = 10; *n* (PCE) = 10; *n* (TNBS) = 6; *n* (Ethanol) = 5; *n* (Sham) = 5; ** *p* < 0.01; *** *p* < 0.001 compared with sham group, one-way ANOVA followed by Tukey’s post hoc test]. Abbreviations: IBD, inflammatory bowel disease; PCE, phenolic compounds extract; TNBS, 2,4,6-Trinitrobenzene sulfonic acid.

**Figure 6:**
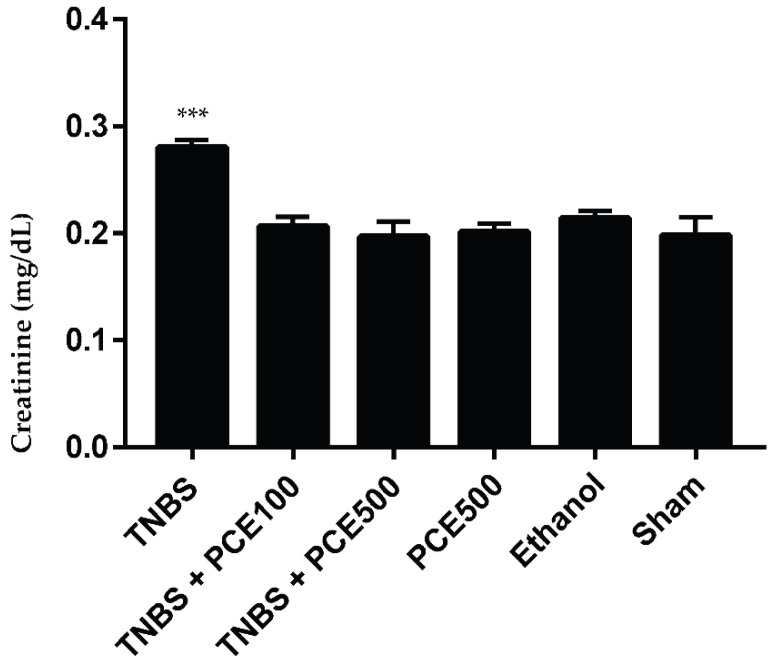
Effect of PCE treatment on creatinine concentration in the IBD. The results are represented as mean ± SD [ *n* (TNBS + PCE100) = 10; *n* (TNBS + PCE500) = 10; *n* (PCE) = 10; *n* (TNBS) = 6; *n* (Ethanol) = 5; *n* (Sham) = 5; *** *p* < 0.001; compared with sham group, one-way ANOVA followed by Tukey’s post hoc test]. Abbreviations: IBD, inflammatory bowel disease; PCE, phenolic compounds extract; TNBS, 2,4,6-Trinitrobenzene sulfonic acid.

The renal function of mice with TNBS-induced colitis was evaluated based on the concentrations of the renal damage markers, such as urea and creatinine (Figure 5 and Figure 6). The TNBS group exhibited a significant increase in urea compared to the sham group (66.8 ± 3.2 vs. 44.7 ± 8.5 mg/dL, *p* < 0.001). Similarly, TNBS group also demonstrated asignificant increase in creatinine levels compared to the sham group (0.28 ± 0.02 vs. 0.20 ± 0.04 mg/dL, *p* < 0.001). The PCE treatment at both dosages promoted a significant decrease of both biochemical markers compared to the TNBS group. However, differences between the urea and creatinine levels in mice treated with PCE were not significant when compared with sham group. The TNBS+PCE100 and TNBS+PCE500 groups revealed 44.7 ± 4.7 and 46.7 ± 6.3 mg/dL of urea and 0.21 ± 0.03 and 0.20 ± 0.03 mg/dL of creatinine, respectively. Among the control groups, the PCE500 group (32.3 ± 3.5 mg/dL) and the sham group showed statistically significant differences in urea levels (*p* < 0.01). While in creatinine levels, there were no statistically significant differences between control groups, such as PCE500, ethanol and sham.

#### 3.3.5. Hepatic Function

**Figure 7:**
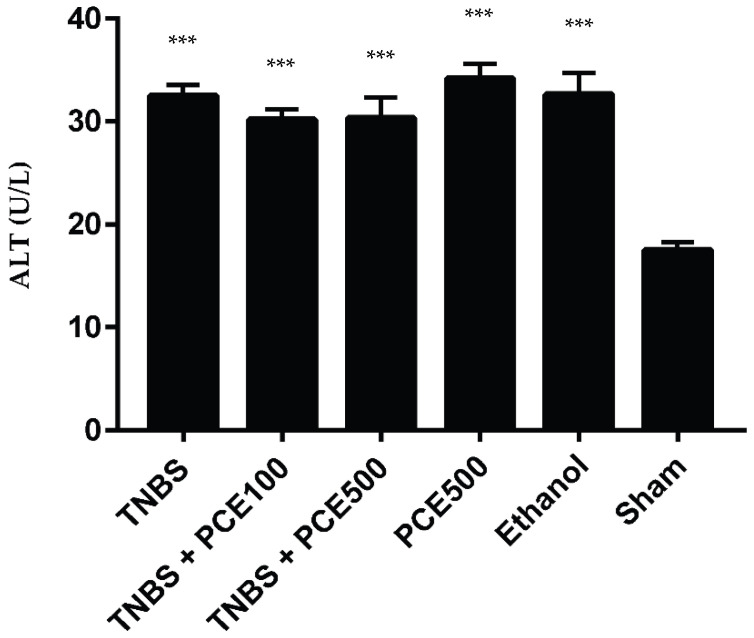
Effect of PCE treatment on ALT concentration in the IBD. The results are represented as mean ± SD [ *n* (TNBS + PCE100) = 10; *n* (TNBS + PCE500) = 10; *n* (PCE) = 10; *n* (TNBS) = 6; *n* (Ethanol) = 5; *n* (Sham) = 5; *** *p* < 0.001compared with sham group, one-way ANOVA followed by Tukey’s post hoc test]. Abbreviations: ALT, alanine aminotransferase; IBD, inflammatory bowel disease; PCE, phenolic compounds extract; TNBS, 2,4,6-Trinitrobenzene sulfonic acid.

The hepatic function was evaluated based on the ALT concentration in the plasma of the mice present in all groups (Figure 7). The TNBS group exhibited a significant increase in ALT concentration when compared with the sham group (32.5 ± 2.5 vs. 17.5 ± 1.8 U/L, *p* < 0.001). The PCE treatment at both dosages did not promote a decrease of ALT levels with statistical significance, compared to the TNBS group. However, the treated mice, present on TNBS+PCE100 and TNBS+PCE500 groups, showed a significant increase in ALT levels (30.2 ± 3.2 and 30.3 ± 6.3 U/L, respectively) relative to the sham group (*p* < 0.001). The PCE500 (34.2 ± 4.4 U/L) and ethanol (32.7 ± 4.7 U/L) groups were significantly higher than the sham group (*p* < 0.001).

### 3.4. Analysis of the Inflammatory Response

**Figure 8:**
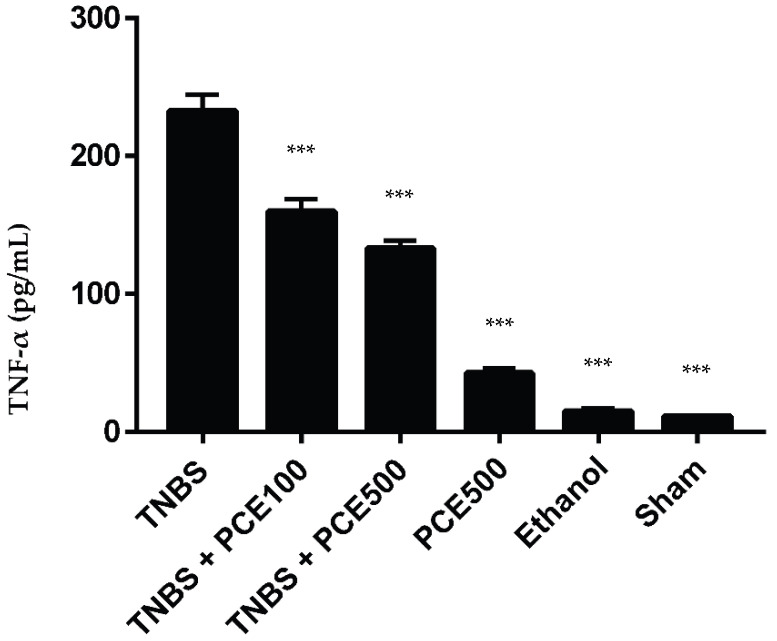
Effect of PCE treatment on TNF-α concentration in the IBD. The results are represented as mean ± SD [*n* (TNBS + PCE100) = 10; *n* (TNBS + PCE500) = 10; *n* (PCE) = 10; *n* (TNBS) = 6; *n* (Ethanol) = 5; *n* (Sham) = 5; *** *p* < 0.001 compared with TNBS group, one-way ANOVA followed by Tukey’s post hoc test]. Abbreviations: IBD, inflammatory bowel disease; PCE, phenolic compounds extract; TNBS, 2,4,6-Trinitrobenzene sulfonic acid; TNF-α, tumor necrosis factor-α.

In pathological conditions, pro-inflammatory cytokines, such as TNF-α, can become dysregulated, promoting an exacerbated inflammatory response (Figure 8). In this case, the TNBS group revealed a significant increase of TNF-α compared to the sham group (232.5 ± 28.9 vs. 11.2 ± 0.5 pg/mL, *p* < 0.001). The treatment with PCE at both dosages, 100mg/kg/day and 500 mg/kg/day, allowed the TNF-α levels to significantly decrease compared to the TNBS group (159.7 ± 29.1 and 133.1 ± 18.0 pg/mL, respectively; *p* < 0.001). The other control groups, PCE500 and ethanol, also demonstrated a significant decrease in this cytokine compared to the TNBS group (42.4 ± 11.7 and 14.9 ± 4.5 cm, respectively; *p* < 0.001).

## 4. Discussion

The role of several foods in disease prevention have been attributed, in part, to the antioxidant properties of their constituent polyphenols. Recent studies have shown that many dietary polyphenolic constituents derived from plants are more effective antioxidants than vitamins E or C [25]. Published data have reported that polyphenols modulate a large number of targets relevant to inflammation and colitis [26]. These compounds modulate the NF-kB pathway, and some of them also inhibit the MAPK cascade and induce nuclear factor (erythroid-derived-2)-like 2. Besides that, polyphenols reduce pro-inflammatory cytokines, induce inflammatory cytokines, inhibit nitric oxide production and inhibit COX-2 activity [26]. Foods rich in resveratrol, curcumin, quercetin and anthocyanin appear to have the most evidence for efficacy and safety in rodent colitis models. In IBD, these compounds have several benefits, namely reduction of bleeding, improvement in stool consistency and histological appearance, decreased weight loss and protection against shortening of the colon [26]

Mice with TNBS-induced colitis were followed daily and monitored in terms of clinical signs. As expected, the TNBS groups revealed an alteration of intestinal motility, characterized by diarrhea or soft stools and severe edema of the anus. These results are compatible with a correct induction of experimental colitis. PCE treatment was able to attenuate diarrhea and moderate edema of the anus compared to non-treated mice. Regarding changes of body weight, the groups treated with PCE did not present significant differences from the TNBS group. In this sense, these compounds did not demonstrate a significant effect on the body weight of the animals. This is in accordance with what is described in the literature because weight gain after the administration of phenolic compounds is rare [26]. Additionally, although the fact that the weight can be an important clinical sign to be aware of, it is not considered a specific and sensitive marker in several cases.

TNBS-induced colitis is characterized by a shortened colon [24]. Indeed, it was possible to confirm this statement because the lowest value obtained for the colon length was from the TNBS group. In fact, the treatment with PCE, at the highest dosage, showed to be beneficial since the values obtained were significantly higher in comparison to the TNBS group.Once again, these results are in agreement with the data currently available, which have reported a protective role of polyphenols on the colon tissue [26].

The determination of fecal hemoglobin allows for the evaluation of the intensity of the hemorrhagic focus and can be useful in the detection of lesions accompanied by bleeding [27]. Taking into account the obtained results, it is possible to confirm a beneficial effect from the administration of both dosages of PCE, in a dose dependent manner, by the significant reduction of the concentration of fecal hemoglobin. Combining the results of the colon length and the presence of fecal hemoglobin, a protection from polyphenols in the colon tissue can be confirmed, which was expected, taking the currently available literature into account [26]. 

The intestinal ALP is expressed on the enterocytes and is responsible for mucosal defense [28]. The TNBS group demonstrated higher ALP levels compared to the other groups, such as PCE500, ethanol and sham groups, suggesting that the increase of this biomarker is due to the induced intestinal lesion. In groups treated with PCE, a significant decrease of ALP levels was observed, compared with the TNBS group. This was probably due to the potential anti-inflammatory effect of the phenolic compounds because the inhibition of the inflammatory process promotes a decrease of the ALP level [1,29]. Indeed, the values obtained for the PCE-treated groups were similar to the control groups, demonstrating that these compounds may have the capability to revert the increased concentration of ALP caused by the induction of colitis. However, there was no dose-dependent effect between the two dosages tested.

To determine whether treatment with PCE may modulate the inflammatory process through the regulation of the secretion of pro-inflammatory cytokines, the colon levels of TNF-α were analyzed. It was observed that the levels of this pro-inflammatory cytokine were significantly reduced after the administration of PCE, confirming the anti-inflammatory effect of polyphenols that has been described in the literature [26]. These results may occur due the fact that phenolic compounds suppress the degradation of the inhibitory IkB protein and, consequently, the NF-kB activation [13,30]. Thus, because NF-kB can induce TNF-α secretion and these compounds inhibit NF-kB activation, a decrease in TNF-α concentration was expected. Among the two dosages tested, there were no statistically significant differences.

The determination of plasma urea, creatinine and ALT levels allows one to understand whether TNBS-induced colitis causes renal and hepatic impairment. Additionally, it is also important to quantify these biomarkers to evaluate the safety profile of the applied treatment, taking into account the renal and hepatic functions. Indeed, the treatment with PCE allowed for the obtaining of better results in these parameters—except for the ALT levels, in which the differences were not significant in relation to the TNBS group. However, there are several publications that have reported the hepatoprotective effects of these compounds. Future studies will be essential to understand the mechanism underlying the beneficial effect of PCE in the extraintestinal manifestations of IBD [31,32]. These results also allow for the conclusion that PCE does not promote renal and/or hepatic changes as an adverse drug reaction in this experimental colitis model.

According to the results obtained in this study, it is possible to observe a beneficial role from the administration of phenolic compounds in a mouse model of colitis, without significant side effects. Indeed, the development and application of natural-based products can be a very promising approach for a safer long-term treatment. In fact, one of the major concerns in terms of the pharmacological treatment in IBD patients is the toxicity associated with chronic utilization. In this sense, the application of natural products, such as the aqueous extract of phenolic compounds evaluated in this study, can be as important of a tool as an adjuvant therapy, which can be translated in the reduction of the exposition to commonly used drugs, by reducing its dose. Additionally, it can also be considered important as a preventive tool of acute crisis because it can be administrated over a longer spectrum of time and has a relatively safe profile, as if it was a supplement. Individuals with several risk factors associated to the development of IBD, such as genetics and environmental variables, can also be a target for the administration of natural-based products, preventing and/or reducing the rate of development of this disease.

Although the administration of an extract of phenolic compounds obtained from red wine had demonstrated an anti-inflammatory effect on a TNBS-induced acute model of colitis, there are some points to be taken in account, such as: the administration route, where the phenolic compounds are commonly consumed orally through alimentation and/or supplementation, passing through several enzymatic reactions on the digestive system and, in this experiment, administered IP, resulting in a possible augmented absorption. Therefore, it would be interesting to evaluate the effect of this extract through an enteral administration in future studies. Additionally, according to the fact that this acute animal model of disease mimics the active phase of IBD in patients, it would be also interesting to evaluate the efficacy and safety of PCE on a chronic TNBS-induced model of colitis. In this sense, it would be possible to investigate its efficacy and safety profiles over an extended period of time. 

## 5. Conclusions

In conclusion, it was possible to observe a beneficial effect of administrating PCE in a TNBS-induced animal model of colitis. Indeed, PCE showed a positive effect in the inflammatory response related to the pathogenesis of IBD through the reduction of TNF-α, a well-known pro-inflammatory cytokine that is present in this disease. Additionally, it demonstrated a beneficial effect on the extraintestinal manifestations, such as the renal function, and did not show any significant adverse effects upon its use. However, the findings related to body weight were not consistent with the remaining evaluated parameters, which shows that the extract might not influence significantly this marker.. In addition, the administration of phenolic compounds in a longer spectrum of time should also be evaluated through the development of a chronic model of colitis, in order to ascertain its efficacy and safety. In sum, according to the results obtained in this experiment, it is possible to conclude that the administration of phenolic compounds can be a very promising approach for the future management of chronic inflammatory diseases, such as IBD.

## Data Availability

Not applicable.
